# Elemental anomalies in the hippocampal formation after repetitive electrical stimulation: an X-ray fluorescence microscopy study

**DOI:** 10.1007/s00775-014-1177-7

**Published:** 2014-07-16

**Authors:** J. Chwiej, H. Gabrys, K. Janeczko, J. Kutorasinska, K. Gzielo-Jurek, K. Matusiak, K. Appel, Z. Setkowicz

**Affiliations:** 1 Faculty of Physics and Applied Computer Science, AGH University of Science and Technology, Krakow, Poland; 2 Department of Neuroanatomy, Institute of Zoology, Jagiellonian University, Krakow, Poland; 3 Deutsches Elektronen-Synchrotron (DESY), Hamburg, Germany

**Keywords:** Rat kindling model of epilepsy, Transauricural electroshocks, Topographic and quantitative elemental analysis, X-ray fluorescence microscopy, Synchrotron radiation

## Abstract

Our previous studies carried out on the pilocarpine model of seizures showed that highly resolved elemental analysis might be very helpful in the investigation of processes involved in the pathogenesis of epilepsy, such as excitotoxicity or mossy fiber sprouting. In this study, the changes in elemental composition that occurred in the hippocampal formation in the electrical kindling model of seizures were examined to determine the mechanisms responsible for the phenomenon of kindling and spontaneous seizure activity that may occur in this animal model. X-ray fluorescence microscopy was applied for topographic and quantitative analysis of selected elements in tissues taken from rats subjected to repetitive transauricular electroshocks (ES) and controls (N). The detailed comparisons were carried out for sectors 1 and 3 of the Ammon’s horn (CA1 and CA3, respectively), the dentate gyrus (DG) and hilus of DG. The obtained results showed only one statistically significant difference between ES and N groups, namely a higher level of Fe was noticed in CA3 region in the kindled animals. However, further analysis of correlations between the elemental levels and quantitative parameters describing electroshock-induced tonic and clonic seizures showed that the areal densities of some elements (Ca, Cu, Zn) strongly depended on the progress of kindling process. The areal density of Cu in CA1 decreased with the cumulative (totaled over 21 stimulation days) intensity and duration of electroshock-induced tonic seizures while Zn level in the hilus of DG was positively correlated with the duration and intensity of both tonic and clonic seizures.

## Introduction

Epilepsy, one of the oldest diseases known to mankind and the most common neurological disorder affecting individuals of all ages, is characterized by recurrent spontaneous seizures [1]. In around 40 % of epilepsy cases, disease etiology is known. The most frequent causes of acquired epilepsies comprise: brain insults including traumatic brain injury, ischemic stroke, intracerebral hemorrhage, infections, tumors, cortical dysplasia, neurodegenerative diseases, and prolonged acute symptomatic seizures [1]. Although people at risk of epilepsy can be identified, there is still no prophylactic treatment that would prevent the development of the disease [2, 3].

It is long known that there is often a seizure-free period between brain insult and clinical manifestation of seizures [4]. During this silent phase, a cascade of poorly understood processes transforms the non-epileptic brain into the one generating spontaneous recurrent seizures. A better recognition of these pathological processes is necessary to identify potential targets for new antiepileptogenic therapies used after brain insults of different types [5].

Many different animal models are used to study processes involved in epileptogenesis. They include: kindling, post-*status epilepticus*, traumatic brain injury, stroke, and febrile seizure models [4, 6–8]. Of these models, kindling and post-*status epilepticus* model of temporal lobe epilepsy (TLE) are recommended for examination of new antiepileptogenic and disease-modifying therapies [6].

Our previous research focused on the mechanisms involved in the pathogenesis and progress of epilepsy in the pilocarpine model [9–13]. To achieve this goal, we applied the experimental techniques based on synchrotron radiation. X-ray fluorescence microscopy was used for analysis of elemental concentrations while Fourier transform infrared microscopy was applied for biochemical analysis of tissues with the spatial resolutions of around 10–15 μm. The obtained results showed that excitotoxicity, mossy fiber sprouting, iron-catalyzed oxidative stress and decreased creatine kinase activity should be taken into account as the mechanisms underlying the neurodegenerative changes of the hippocampal formation and spontaneous seizure activity in the chronic phase of pilocarpine model [9–13]. The present study addresses the above question using electrical stimulation as a seizuregenic agent because it is devoid of neurochemical specificity and, therefore, shows no functional preference for any neuronal subsystem. This appears to be the most unbiased approach.

In the present study, anomalies of elemental concentrations in the hippocampal formation in the electroshock kindling rat model of epilepsy were examined. Kindling was first described by Goddard et al. in 1969 and is defined as repeated, usually electrical, stimulation of the brain which initially induces only subclinical after discharges, but over time after many repetitions leads to a progressive increase in the severity and duration of seizures [14, 15]. Stimulation of a specific brain region, such as the amygdala or hippocampus, is usually carried out through chronically implanted depth electrodes [8]. However, brain injury caused by electrode implantation may play a significant role in the kindling process (prokindling effect), leading to epileptiform potentials in the hippocampus and inducing changes in elemental concentrations in the brain regions involved in epilepsy pathogenesis and progression [8, 16–18]. Therefore, in the present study, we used repetitive electrical stimulation through ear-clip electrodes.

X-ray fluorescence microscopy was used to investigate the distribution and accumulation of elements in brain tissues taken from rats subjected to repetitive transauricular electroshocks and from naive controls. Because trace elements are involved in many processes that may be implicated in the pathogenesis and progress of epilepsy, the analysis of their tissue levels and distributions may shed some new light on the kindling phenomenon which is still not fully understood.

## Materials and methods

All animal use procedures were approved by the Bioethical Commission of the Jagiellonian University and were in agreement with the international standards. Adult Wistar rats were obtained from an animal colony of the Department of Neuroanatomy, Institute of Zoology, Jagiellonian University, Krakow. During their whole life the animals were maintained under conditions of controlled temperature (20 ± 2 °C) and illumination (12-h light:12-h dark cycle). A solid diet (Labofeed) and water were available ad libitum.

### Two groups of animals were examined:

N–six naive control animals which on the day 80th of postnatal development were perfused with physiological saline solution of high analytical purity;

ES–twenty male rats which from the day 60th of their postnatal development were daily (during 21 stimulation days) subjected to transauricural electroshocks through a pair of ear-clip electrodes. Sinusoidal sub-maximal current of 10 mA and frequency of 60 Hz were produced by a Rodent Shocker RS type 221 (Hugo Sachs Elektronik–Harvard Apparatus GmbH, Germany). The duration of electrical stimulation was 1 s. The intensity of electrical current and the duration of stimulation were determined based on preliminary experiments on intact rats. The highest values of the parameters that did not induce seizures of maximal severity (tonic extension of the hindlimbs) were chosen.

During and after stimulation the animals were observed until electroshock-induced behavioral symptoms disappeared and rats regained their normal posture. The animal behavior was video recorded for further assessments. Tonic and clonic seizures were distinguished and evaluated using separate scales. The tonic seizures were evaluated according to the severity index scale previously established for electroshock-induced seizures [19–21]: 0—no seizure, 1—forelimb extension without hind limb extension, 2—complete forelimb extension and partial hind limb extension, 3—complete (parallel to the tail) hind limb extension.

Evaluation of clonic seizures was performed using a modified 3-point version of the Racine’s limbic seizure scale [15] previously used in our studies on pilocarpine-induced seizures [22].

After 21 stimulation days, the animals subjected to electroshocks and age matched controls were deeply anesthetized using Morbital (Biowet) and perfused with 0.9 % saline of high analytical grade. The brains removed from the skulls were first deeply frozen in liquid nitrogen and then cut horizontally in a cryomicrotome into 12-μm thick sections. The specimens of the dorsal part of the hippocampus [23] were mounted on Ultralene^®^ foil and freeze-dried.

## Experimental method and apparatus

X-ray fluorescence microscopy was used for the qualitative, quantitative and topographic elemental analysis and the measurements were carried out at HASYLAB beamline L. The polycapillary optics was applied to focus the 17 keV beam to the final focal spot of 15 micrometers in diameter. Silicon drift detector positioned at the angle of 45° in respect to the sample and 90° in respect to the exciting beam was used to detect the fluorescence radiation from the samples which were mapped in two dimensions. The time of single fluorescence spectrum acquisition was 12 s.

For spectrometer calibration and calculations of elemental sensitivities, MICROMATTER XRF calibration standards (GaP, KCl, CaF_2_, Fe, ZnTe, Se and RbI) were used.

## Results

Twelve-micrometer thick freeze-dried tissue slices were examined in this study. According to Szczerbowska-Boruchowska, such samples can be regarded as thin in quantitative analysis using X-ray fluorescence for all the analyzed elements. For thin samples, intensity of characteristic X-ray radiation depends linearly on the concentration of the element in the sample and it opens the opportunity to use the external standard method for quantification by measuring thin reference materials [24].

**Fig. 1:**
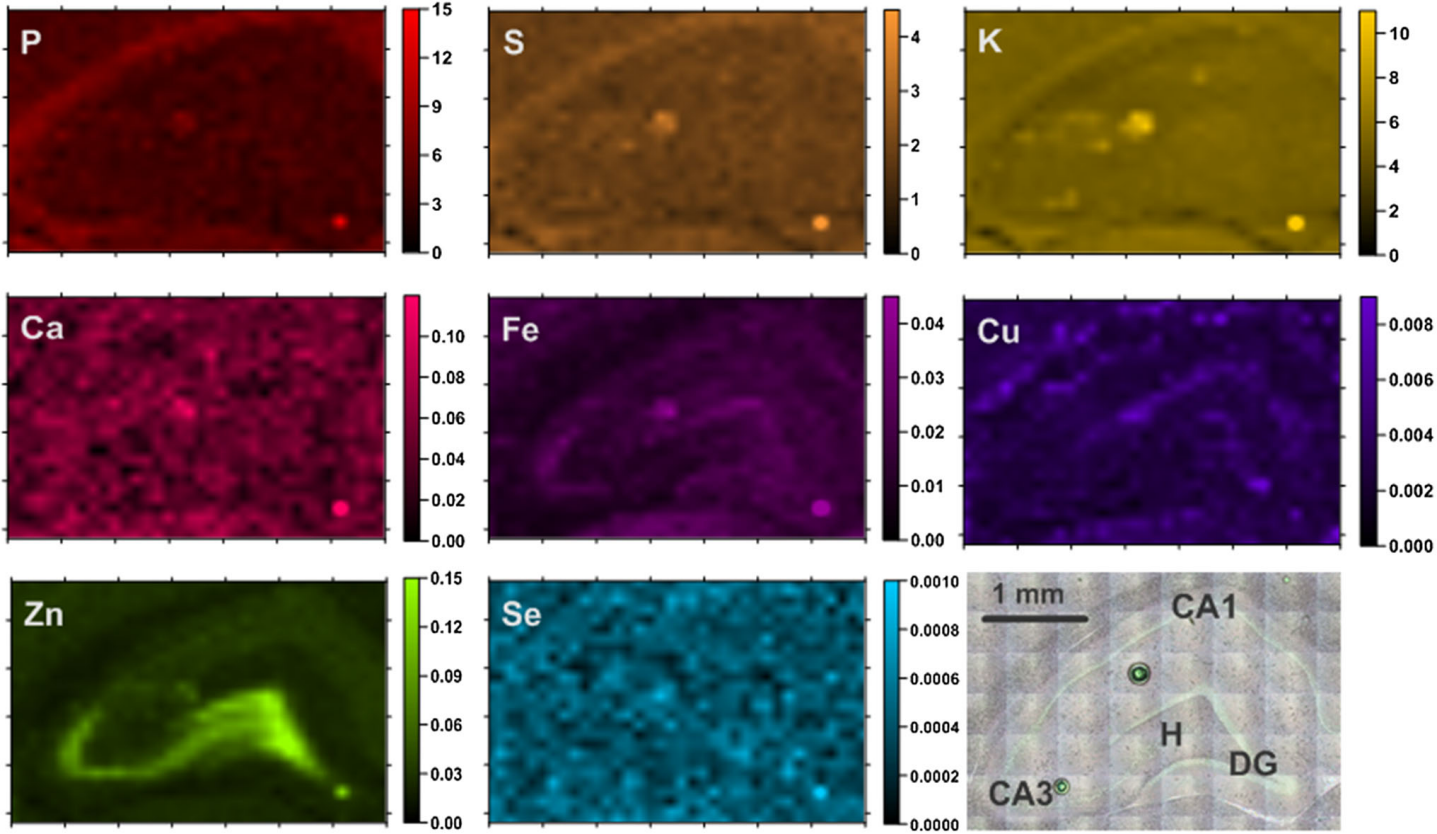
Elemental maps of the hippocampal formation from a kindled animal. Scales display areal densities of the elements in μg/cm^2^

**Fig. 2:**
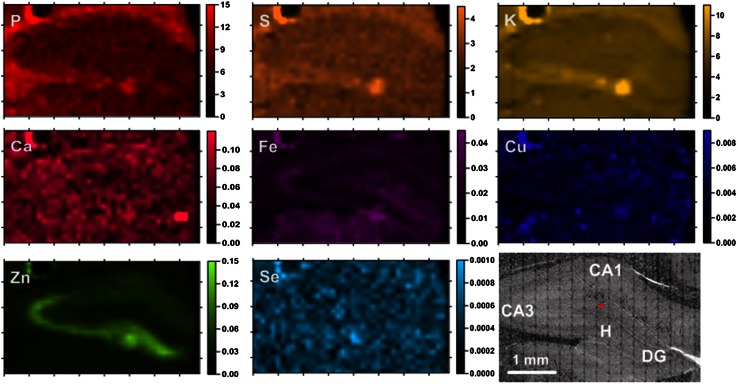
Elemental maps of the hippocampal formation from a control animal. Scales display areal densities of the elements in μg/cm^2^

The analysis of single X-ray fluorescence spectra as well as batch processing of large data sets was carried out using PyMCA software freely available for non-commercial use. The obtained net peak areas of K-α lines of the analyzed elements and elemental sensitivities evaluated based on measurements of calibration standards were used to calculate elemental areal densities for the examined tissue points. Areal densities (*M*_T_) were calculated according to Eq. 1:

1 $$M_{\text{T}} = \frac{{Y_{\text{T}} }}{{t_{\text{T}} I_{\text{T}} S}}$$

where, *Y*_T_ is the net peak area of the measured element for the tissue sample, *t*_T_ is the measurement time for the tissue sample, *I*_T_ is the incident photon flux for the tissue sample, *S* is the sensitivity for the measured element, given by Eq. 2:

2 $$S = \frac{{Y_{\text{S}} }}{{t_{\text{S}} I_{\text{S}} M_{\text{S}} }}$$

where, *Y*_S_ is the net peak area of the measured element for the standard sample, *t*_S_ is the measurement time for the standard sample, *I*_S_ is the incident photon flux for the standard sample, *M*_S_ is the areal density of the measured element in the standard sample. The first step of the study was the topographic analysis of the distributions of: P, S, K, Ca, Fe, Cu, Zn and Se in the analyzed slices of hippocampal formations. Figures 1, 2 show examples of elemental maps obtained for kindled and control rats, respectively. In addition, the microscopic pictures of the scanned tissue areas are presented.

**Fig. 3:**
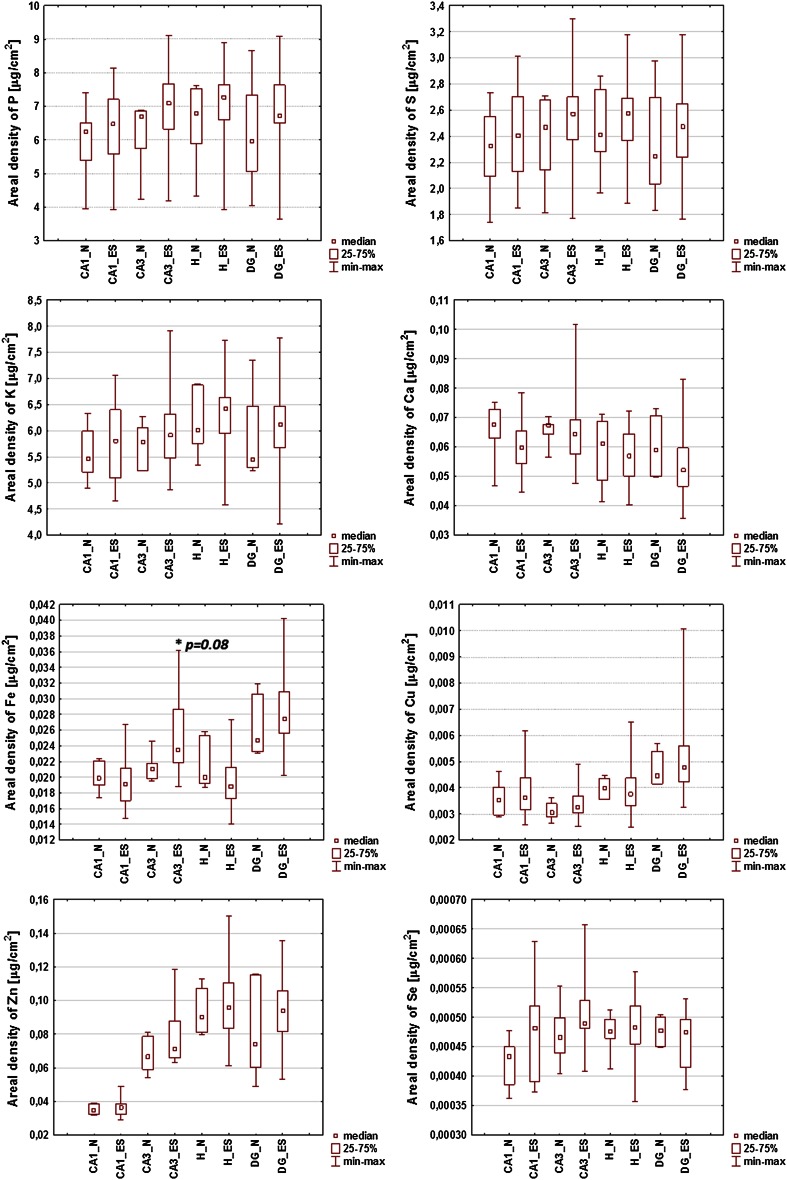
Comparison of median, minimal and maximal values of areal densities of elements in the analyzed hippocampal areas (CA1, CA3, DG and H). Statistically significant differences between ES and N groups were marked with *asterisk* followed by *p* value of *U* Mann–Whitney test

For all examined samples the mean elemental areal densities were calculated for sectors 1 and 3 of the Ammon’s horn (CA1 and CA3, respectively), the dentate gyrus (DG) and the hilus of DG (H). The localization of the above-mentioned areas within the hippocampal formation can be seen in Figs. 1, 2. In order to compare the kindled and control animals and evaluate the statistical significance of the differences in elemental composition between them, the medians for ES and N groups of rats were used and the obtained results are presented in Fig. 3.

**Fig. 4:**
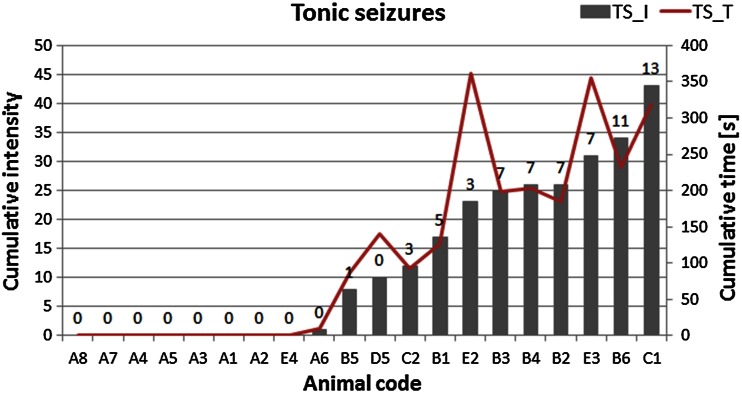
Cumulative (totaled over 21 stimulation days) intensity (TS_I) and cumulative duration (TS_T) of tonic seizures. The numbers above the *bars* signify the numbers of seizures of maximal intensity occurring during the 21-day stimulation period

**Fig. 5:**
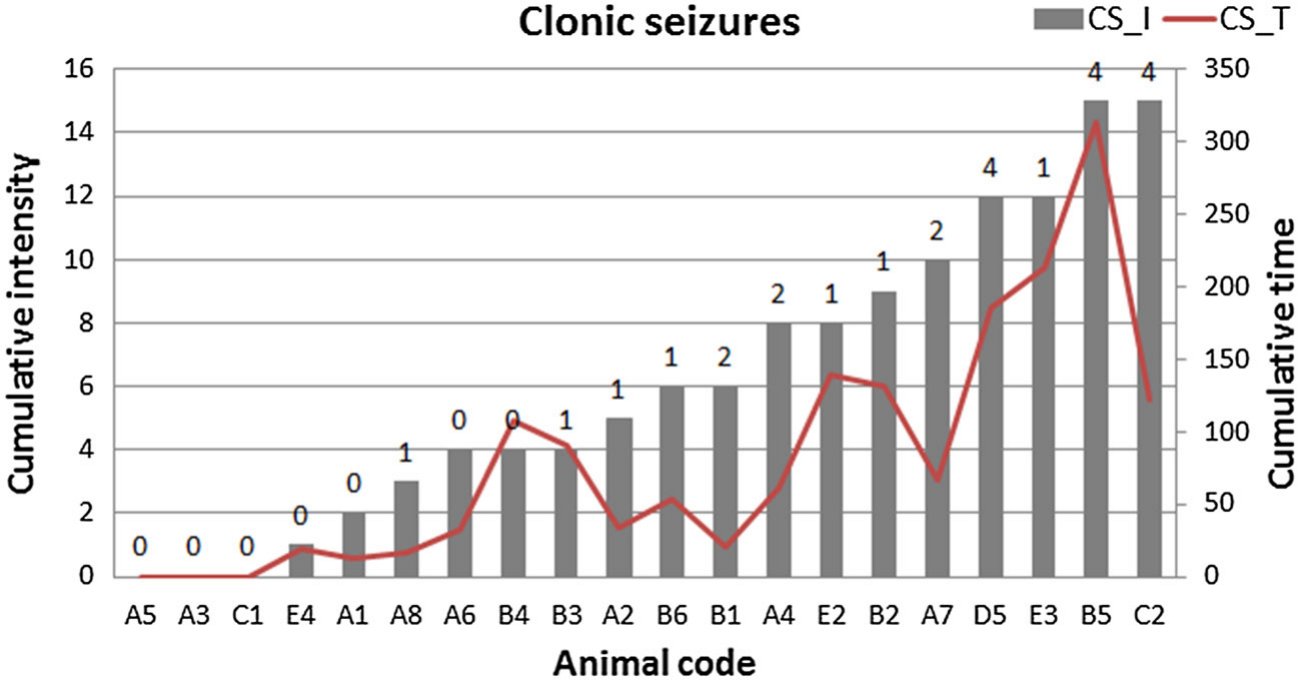
Cumulative (summed for 21 stimulation days) intensity (CS_I) and cumulative duration (CS_T) of clonic seizures. The numbers above the *bars* signify the number of seizures of maximal intensity occurring during the 21-day stimulation period

Statistical significance of differences between median values obtained for ES and N groups was evaluated using the non-parametric *U* Mann–Whitney test at the confidence level of 90 %. As can be seen in Fig. 3, the increased Fe level within the area of CA3 was the only significant change noticed in the kindled animals. This result is not surprising when we take into account a wide variability of the susceptibility of animals to electroshocks as evaluated based on cumulative seizure intensity and duration and presented in Figs. 4, 5. Figure 4 shows the cumulative intensity and duration of tonic seizures, and the number of tonic seizures of maximal intensity (recorded during 21-day stimulation period) for all animals subjected to electroshocks. Analogous data for clonic seizures are shown in Fig. 5. The cumulative intensities and durations of seizures were calculated as the sums of these parameters recorded on every of 21 stimulation days.

The next step of the study was to analyze relationships between elemental areal densities and parameters describing electroshock-induced changes in animal behavior. Among behavioral data, the cumulative intensity and duration of tonic and clonic seizures were chosen for the correlation analysis. The relationships between the elemental areal densities measured using X-ray fluorescence microscopy and cumulative behavioral parameters were evaluated based on Spearman’s rank correlation coefficients (*r*_s_) at the significance level of 0.1. All the calculated *r*_s_ values are presented in Table 1 and statistically significant data are highlighted in bold.

**Table 1:** The values of Spearman’s rank correlation coefficients between the elemental areal densities and cumulative behavioral parameters

Area	Parameter^a^	P	S	K	Ca	Fe	Cu	Zn	Se
CA1	TS_I	0.25	0.19	0.20	−0.23	−0.19	**−0.41** ^b^	0.06	0.24
	TS_T	0.13	0.07	0.10	−0.27	−0.21	**−0.49**	−0.03	0.13
	CS_I	0.25	0.09	0.19	0.01	0.25	0.05	0.02	0.16
	CS_T	0.06	−0.04	0.03	−0.13	0.09	−0.11	−0.10	0.05
CA3	TS_I	0.04	0.13	0.19	−0.31	0.14	0.03	0.04	−0.31
	TS_T	−0.12	−0.04	0.02	**−0.39**	0.02	−0.11	−0.02	−0.37
	CS_I	−0.18	−0.02	−0.12	0.16	0.14	−0.22	0.00	0.00
	CS_T	−0.20	−0.08	−0.08	0.01	0.15	−0.22	0.13	−0.07
DG	TS_I	−0.21	−0.11	−0.18	−0.2	−0.17	−0.35	0.18	−0.17
	TS_T	−0.25	−0.17	−0.23	−0.25	−0.21	−0.34	0.14	−0.08
	CS_I	0.09	0.07	0.06	0.09	−0.04	−0.09	0.31	−0.30
	CS_T	0.13	0.12	0.07	0.07	0.01	−0.09	0.21	−0.18
H	TS_I	−0.17	0.03	−0.03	−0.20	0.17	0.18	**0.43**	0.04
	TS_T	−0.24	−0.07	−0.12	−0.26	0.11	0.08	**0.48**	0.00
	CS_I	0.01	0.07	0.12	0.09	0.17	0.06	0.31	0.11
	CS_T	−0.03	0.04	0.07	0.01	0.23	0.15	**0.46**	0.13

^a^TS_I and TS_T cumulative intensity and duration (time) of tonic seizures, CS_I and CS_T cumulative intensity and duration of clonic seizures

^b^The correlations significant at the confidence level of 90 % are highlighted in bold

As can be seen in Table 1, the areal density of Cu in CA1 area decreased with the increased cumulative intensity and duration of tonic seizures. The opposite relationship was observed for Zn in the hilus. In addition, the level of this element in the hilus was positively correlated with cumulative duration of clonic seizures.

Because the results of correlation analysis showed the existence of statistically significant relationships between elemental and behavioral data, in the next step the animals subjected to electrical stimulations were divided into subgroups based on the results of behavioral observations. For this purpose, the cluster analysis was applied. The cluster analysis is the collection of different algorithms and methods for grouping objects of similar kind into respective categories. This data analysis tool allows for sorting different objects into groups in the way that the degree of association between two objects is maximal if they belong to the same group and minimal otherwise [25].

**Fig. 6:**
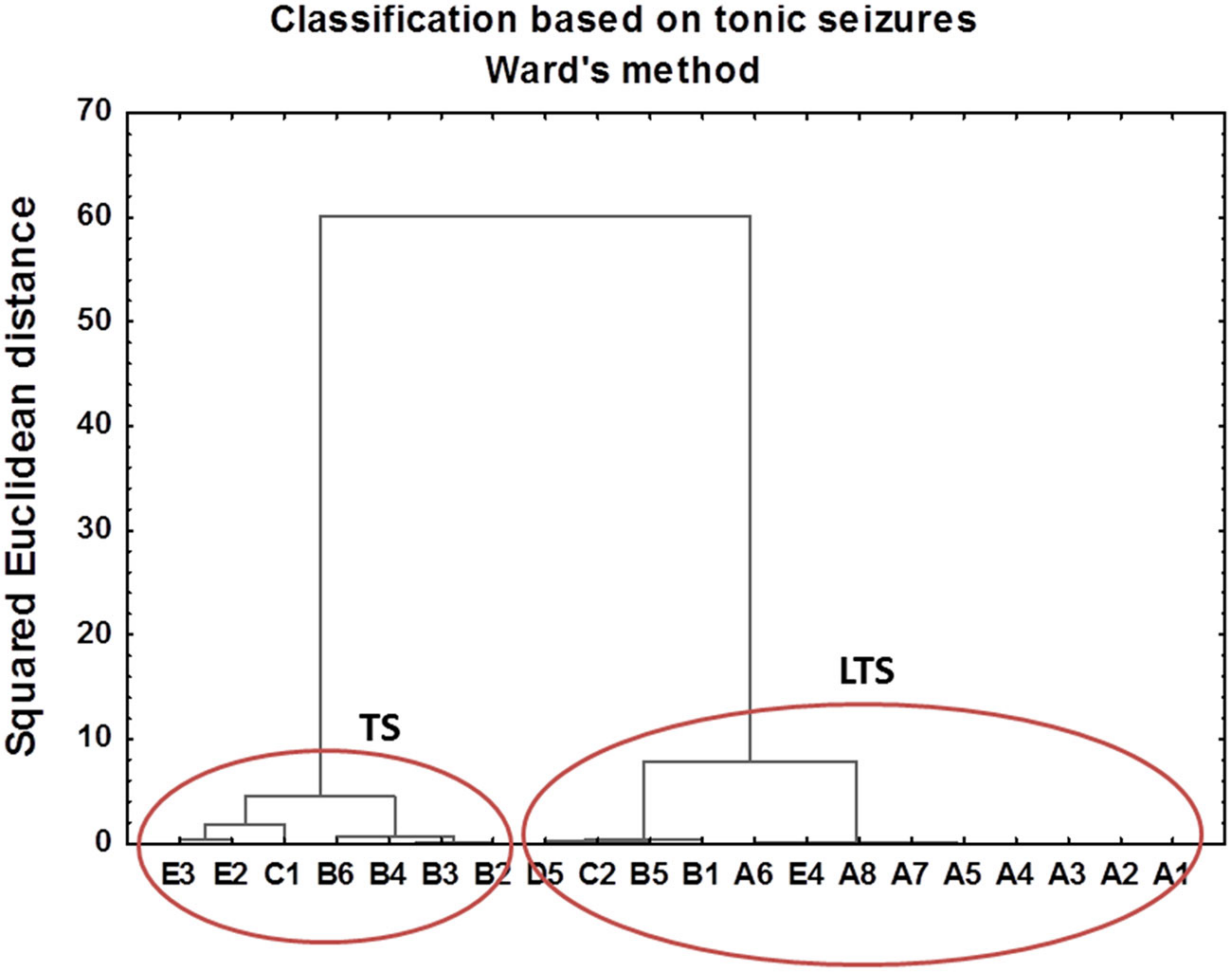
The hierarchical tree plot obtained using Ward’s method for kindled animals. The squared Euclidean distances used as a measure of similarity between observations were calculated based on cumulative parameters describing electroshock-induced tonic seizures

**Fig. 7:**
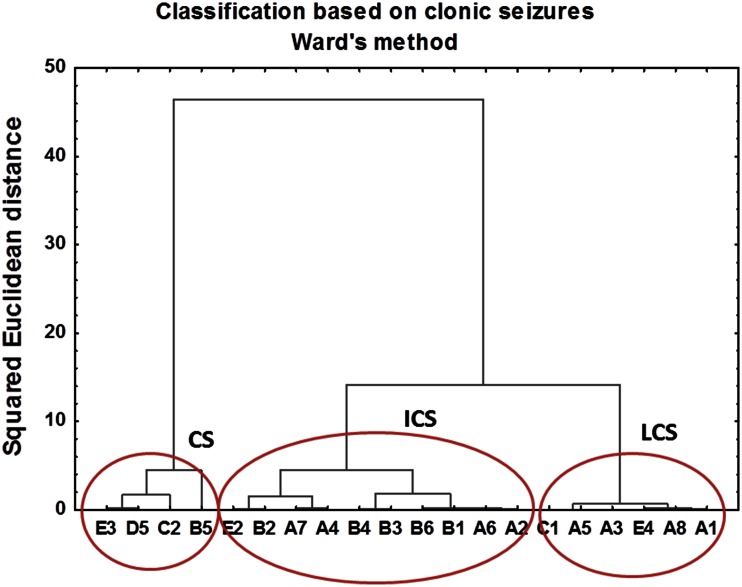
The hierarchical tree plot obtained using Ward’s method for animals subjected to electroshocks. The squared Euclidean distances used as a measure of similarity between observations were calculated based on cumulative parameters describing electroshock-induced clonic seizures

In this study, cluster analysis was used for classification of kindled animals into subgroups based on the cumulative seizure intensity and duration. For non-supervised classification, Ward’s method with squared Euclidean distance as a measure of similarity between cases was applied. This very efficient hierarchical method uses analysis of variance approach to evaluate the distances between clusters and attempts to minimize the sum of squares of any two clusters that can be formed at each step [26]. The results of clustering obtained with Ward’s method are presented as hierarchical tree plots in Figs. 6, 7.

As can be seen in Figs. 6, 7, hierarchical trees obtained for tonic and clonic seizures present different classification patterns. Based on the behavioral parameters describing tonic seizures, animals were divided into two clusters. The first cluster contained animals in which transauricural electroshocks did not induce any or induce only very weak changes in the behavioral parameters used for evaluation of tonic seizures (LTS). Within the second cluster, rats presenting tonic seizures of high intensity and duration were classified (TS).

Based on the cumulative intensities and durations of clonic seizures, three clusters were distinguished using Ward’s method. In addition to the clusters containing the animals presenting low (LCS) or severe (CS) electroshock-induced clonic seizures, an intermediate group (ICS) was also extracted.

The results obtained using Ward’s method were confirmed using *k*-means clustering. In this method, we start with *k* random clusters and move objects between them in order to minimize within group and maximize between group variability [25]. The results of *k*-means clustering, with two clusters distinguished for tonic and three for clonic seizures, confirmed the classification pattern obtained using Ward’s method.

**Fig. 8:**
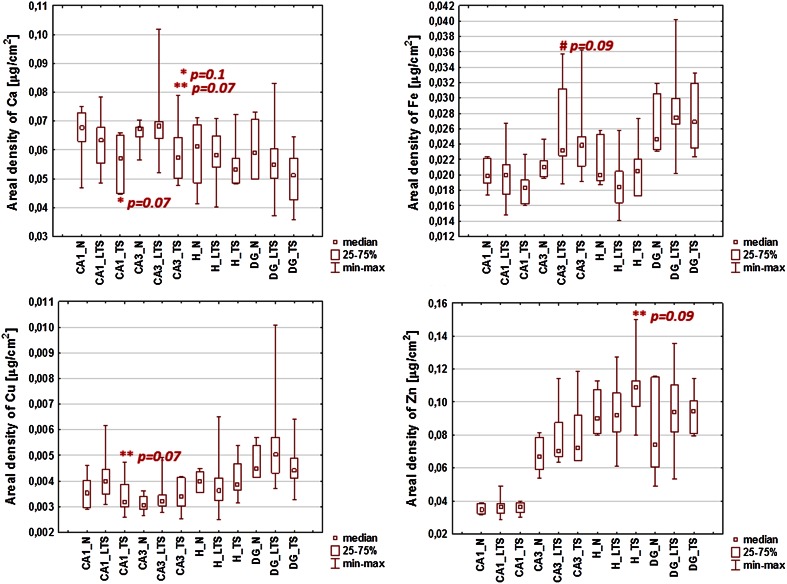
The comparison of medians, minimal and maximal values of areal densities of elements in the analyzed hippocampal areas (CA1, CA3, DG and H) between TS, LTS and N groups. Statistically significant differences: * TS vs. N group, ** TS vs. LTS group, # LTS vs. N group. The marks are followed by *p* values of *U* Mann–Whitney test

**Fig. 9:**
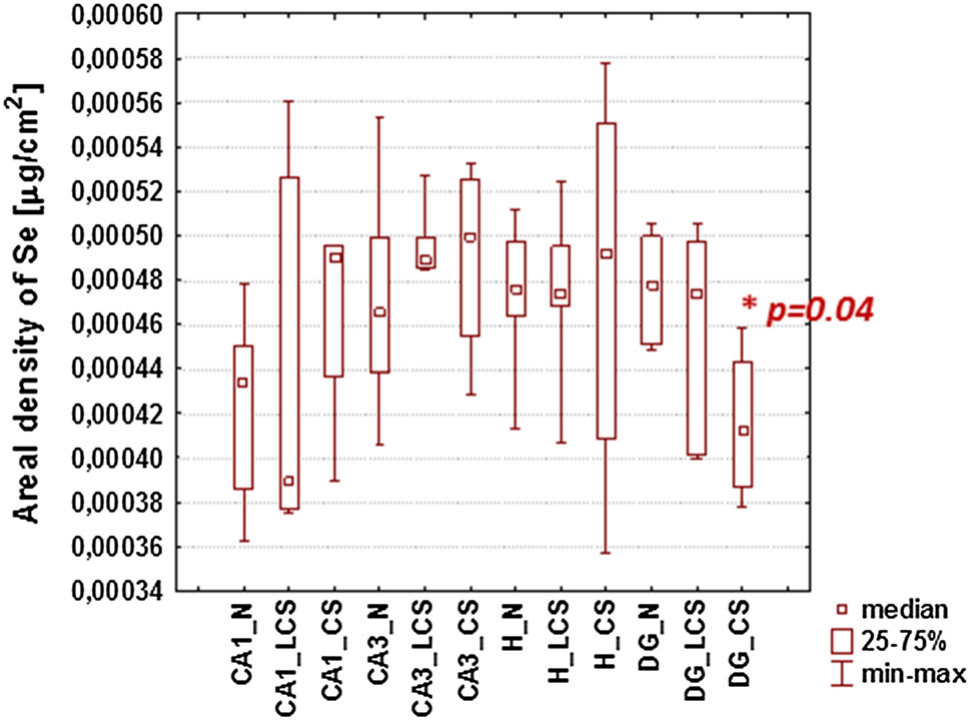
The comparison of medians, minimal and maximal values of Se areal densities in the analyzed hippocampal areas (CA1, CA3, DG and H) between CS, LCS and N groups. A statistically significant difference in Se accumulation was found between CS and control groups and marked with *asterisk* followed by *p* value of *U* Mann–Whitney test

In the next step of the study, the median values of elemental areal densities evaluated for TS, LTS and control groups as well as for CS, LCS and N groups were compared and statistical significance of the differences was tested using *U* Mann–Whitney test. Figures 8, 9 show the box plots obtained for the elements which differed significantly (at the significance level of 0.1) between the analyzed groups. Statistically significant differences for TS and CS groups compared to controls were marked with an asterisk, between TS and LTS groups with double asterisks, while between LTS and N groups with a hash mark.

Comparison of animals presenting severe (TS) and weak or none (LTS) tonic seizures in response to repetitive electrical stimulation showed a few statistically significant differences. A lower levels of Ca in CA3 (*p* value = 0.07) and Cu in CA1 (*p* value = 0.07) hippocampal areas were observed for TS compared to LTS group. The opposite relation was found for Zn in H (*p* value = 0.09). In addition, as can be seen in Fig. 8, TS animals presented lower Ca areal densities vs. controls in CA1 (*p* value = 0.07) and CA3 (*p* value = 0.10) areas. The comparison of LTS and normal rats showed higher Fe level for the first group (*p* value = 0.09).

The only statistically significant difference noticed for subgroups distinguished based on behavioral parameters describing clonic seizures concerned the level of Se in DG area. The areal density of this elements was significantly lower (*p* value = 0.04) in rats in which electroshock-induced clonic seizures occurred.

## Discussion

In the present study, X-ray fluorescence microscopy was applied to analyze the changes in the distribution and accumulation of selected elements, occurring in the rat hippocampal formation as a result of repetitive electrical stimulation. Two groups of animals were examined in this study, namely, adult rats subjected to repetitive transauricural electroshocks through a pair of ear-clip electrodes were compared with naive controls. Electrical stimulation differs radically from any systemically administered seizurogenic pharmacologic agents [21] because it is short-lasting and has precisely defined time of action. This is why the effects of electroshocks cannot be directly compared with those induced by pilocarpine or kainic acid injections (post-*status epilepticus* models). Electroshock-induced tonic or clonic seizures had different functional relationships between the two types of response, hypothetically having different anatomical substrates [27] that should be the subject of a detailed elemental analysis.

The quantitative elemental analysis was performed for CA1, CA3, DG and H of the hippocampal formation. Statistical significance of differences in elemental areal densities between electrically stimulated and control rats and between subgroups of kindled animals was verified using non-parametric *U* Mann–Whitney test. The comparison of ES and N groups showed only one statistically significant difference in elemental accumulation, viz. a higher level of Fe was found in CA3 areas from animals subjected to electrical stimulation (*p* value = 0.08). An increased level of Fe in the kindled group might suggest an important role of Fe-catalyzed oxidative stress in the pathogenesis of kindling-induced epilepsy, like it was previously observed for the pilocarpine model of TLE [11].

The elevated Fe level may be the results of an increased ferritin (an iron-storage protein) expression which, according to the work of Gorter et al. [28] occurs in epileptic rats in specific cells (microglia) from cortical layers of the parahippocampal region. By catalyzing Fenton’s reaction, Fe may lead to an increased production of reactive oxygen species which, then, may induce a cascade of cellular and molecular changes leading to hyperexcitability of neurons or to neuronal death [29].

However, this conclusion is in contradiction with further results obtained in this study, when post-kindling cumulative seizure intensity and duration were analyzed in detail. Namely, a statistically significant increase in Fe accumulation was found only in kindled animals in which weak tonic seizures were observed what would rather suggest neuroprotective than neurodegenerative action of this element in the examined animal model. Moreover, we did not notice any correlations between the areal density of Fe and cumulative intensity and duration of both tonic and clonic seizures.

In the next step of the study, animals subjected to electrical stimulation were divided into subgroups containing rats in which kindling phenomenon was or was not observed. For this purpose cluster analysis was used and classification was based on the cumulative intensity and duration of clonic and tonic seizures occurring in animals after electrical stimulation. Hierarchical tree plots obtained using Ward’s method showed different classification patterns for tonic and clonic seizures. It has been hypothesized that transauricular electroshocks, at the initial stage of kindling at least, can activate exclusively brain stem structures [21, 30] what results in tonic seizures. As kindling develops, prosencephalic areas, especially the limbic system, are gradually stimulated to generate clonic seizures. Thus, the involvement of different brain regions in different types of seizures may explain the differences in the patterns of changes in areal metal contents specific to the tonic and clonic convulsions.

Two subgroups of kindled animals (TS and LTS) were distinguished using Ward’s method based on cumulative intensity and duration of tonic seizures. For clonic seizures three subgroups were educed: CS, ICS and LCS. Further statistical analysis using *U* Mann–Whitney tests showed that the levels of Ca in CA1 (*p* value = 0.07) and CA3 (*p* value = 0.10) areas were lower in rats from TS compared to control group. Moreover, TS animals presented lower areal densities of Ca in CA3 (*p* value = 0.07) compared to LTS group what is in agreement with the values of Spearman’s rank correlation coefficients which showed significant negative correlation between the level of this element in CA3 and cumulative duration of tonic seizures. These results are in contrast to those reported previously for the pilocarpine model [9–11]. The areal density of Ca in pilocarpine-treated rats increased significantly a few hours after proconvulsive agent administration. Moreover, for all analyzed hippocampal areas the level of Ca remained elevated for many days after pilocarpine injection what confirmed the hypotheses concerning the engagement of the excitotoxicity in the pathogenesis and progress of spontaneous recurrent seizures in that animal model of TLE [9–11].

Excitotoxicity means excessive or prolonged stimulation of the glutamate receptors which may lead to neuronal hyperactivation and damage. This pathological process contributes to many central nervous system diseases including ischemia, trauma and epilepsy and Ca^2+^ ions are the key mediators of excitotoxic injury [31–33].

*Status epilepticus* is a common condition increasing glutamate release thus augmenting also the risk of excitotoxicity [34]. The elevated extracellular glutamate concentration following seizures increases the Ca^2+^ influx into cells. Then, dysregulation of cellular Ca^2+^ homeostasis results in the activation of a number of enzymes, including phospholipases, endonucleases, and proteases which finally leads to neuronal injury or death [34, 35].

The results obtained for animals subjected to repetitive electrical stimulation did not show increased Ca levels in the examined areas of the hippocampal formation. They also did not confirm the involvement of excitotoxic processes in the hippocampal neurodegeneration in the electrical kindling model of seizures used in the present study. However, areal densities of Ca which were observed in rats subjected to electroshocks compared to controls may suggest the outflow of Ca ions to other brain regions where excitotoxic processes may occur.

The rats with electroshocks induced severe tonic seizures presented higher areal densities of Zn in the hilus of the DG compared to animals that responded to electroshocks with low-intensity tonic seizures (*p* value = 0.09). Moreover, in this hippocampal area, statistically significant positive correlations were found between Zn level and seizure intensity and duration after electrical stimulation. Zn levels in the hilus increased with the cumulative intensity and duration of tonic seizures and with the total duration of clonic seizures. Thus, the obtained relationships suggest that the increased Zn accumulation is not a result of the electrical stimulations themselves but rather an effect of subsequent seizure activity.

The elevated levels of Zn are likely connected with the phenomenon of mossy fiber sprouting which involves the formation of new asymmetrical synaptic contacts between mossy fiber (groups of granule cells axons) terminals, dendrites of granule cells and inhibitory interneurons in the inner molecular layer of the DG [36–38]. An increased Zn level in the hilus of the DG may be an indicator of this process, what results from the fact that large terminals of mossy fibers contain the highest amounts of this element in the brain.

The process of mossy fiber sprouting was previously observed both in the human temporal lobe epilepsy and in different experimental models of this disease [39–42]. Also kindling elicits mossy fiber sprouting and functional synaptogenesis in the supragranular layer, hilus of DG and CA3 region of the hippocampus [39, 43, 44]. The results obtained for kindling evoked by perforant path, amygdala, or olfactory bulb stimulation showed development of mossy fiber synaptic terminals in the supragranular region of DG already 4 days after the initiation of stimulation. The alterations in the terminal projections of the mossy fiber pathway progressed with the evolution of kindled seizures and were observed even 8 months after the last stimulation [44]. Moreover, Cavazos et al. [44] demonstrated that sprouting and synaptic reorganization of mossy fibers strongly correlated with the development, progression, and permanence of the kindling.

The increased Zn level in the areas specific for mossy fiber sprouting was also observed in our previous investigation focused on the elemental changes occurring as a result of pilocarpine-induced seizures [10, 11]. The elevated areal densities of Zn vs. control were found in pilocarpine-treated animals in the phase of stabilization of animal behavior and EEG activity. Like in this study, the levels of Zn correlated with the quantitative parameters describing animal behavior in the acute phase of pilocarpine-induced *status epilepticus*. Therefore, mossy fiber sprouting seems to be the common pathological mechanism of epileptogenesis in pilocarpine and electrical kindling model of seizures.

The analysis of the cumulative intensity and duration of clonic seizures showed only one statistically significant difference between the examined animal groups. Namely, in rats responding to electroshocks with severe clonic seizures, the accumulation of Se in the DG was lower compared to controls (*p* value = 0.04). This result may reflect the neuroprotective action of this element in this type of seizures. Se is a component of glutathione peroxidases, the main biological role of which consists in protecting the organism from oxidative damage [45].

## Conclusions

The results presented in this paper confirmed that animal studies aimed at searching for new neuroprotective and antiepileptic drugs should be based on determination of the processes that may be involved in the pathogenesis of neurodegenerative changes and spontaneous seizure activity. Highly resolved elemental analysis of hippocampal formation may be very helpful in such study. Analyzing the changes in Ca content, we are able to follow the areas of excitotoxic injury, while examining the level of Zn in specific hippocampal areas we can verify the presence of mossy fiber sprouting. In the present study, X-ray fluorescence microscopy was used for the analysis of the changes in elemental accumulation occurring in the rat hippocampal formation as a result of repetitive transauricular electroshocks. The comparison of animals subjected to electrical stimulations, their subgroups determined based on the behavioral observations and controls showed increased Zn levels in the hilus of the DG of rats in which severe electroshock-induced tonic seizures were observed. The changes in the hilar Zn accumulation were positively correlated with the cumulative seizure intensity and duration after electrical stimulation. Therefore, in the light of the obtained results, mossy fiber synaptic reorganization should be taken into account as one of the processes responsible for the appearance of the kindling phenomenon. The hippocampal levels of Ca were not elevated in the animals subjected to repetitive electroshocks which suggests that excitotoxic injury is not responsible for the pathological changes of the hippocampal formation in the electrical kindling model of seizures.
